# Gene Therapeutic Approaches for the Treatment of Mitochondrial Dysfunction in Parkinson’s Disease

**DOI:** 10.3390/genes12111840

**Published:** 2021-11-22

**Authors:** Jannik Prasuhn, Norbert Brüggemann

**Affiliations:** 1 Institute of Neurogenetics, University of Lübeck, 23562 Lübeck, Germany; jannik.prasuhn@neuro.uni-luebeck.de; 2 Department of Neurology, University Medical Center Schleswig-Holstein, Campus Lübeck, 23562 Lübeck, Germany; 3 Center for Brain, Behavior and Metabolism, University of Lübeck, 23562 Lübeck, Germany

**Keywords:** Parkinson’s disease, gene therapy, mitochondria, genome editing

## Abstract

Background: Mitochondrial dysfunction has been identified as a pathophysiological hallmark of disease onset and progression in patients with Parkinsonian disorders. Besides the overall emergence of gene therapies in treating these patients, this highly relevant molecular concept has not yet been defined as a target for gene therapeutic approaches. Methods: This narrative review will discuss the experimental evidence suggesting mitochondrial dysfunction as a viable treatment target in patients with monogenic and idiopathic Parkinson’s disease. In addition, we will focus on general treatment strategies and crucial challenges which need to be overcome. Results: Our current understanding of mitochondrial biology in parkinsonian disorders opens up the avenue for viable treatment strategies in Parkinsonian disorders. Insights can be obtained from primary mitochondrial diseases. However, substantial knowledge gaps and unique challenges of mitochondria-targeted gene therapies need to be addressed to provide innovative treatments in the future. Conclusions: Mitochondria-targeted gene therapies are a potential strategy to improve an important primary disease mechanism in Parkinsonian disorders. However, further studies are needed to address the unique design challenges for mitochondria-targeted gene therapies.

## 1. Introduction

### 1.1. Mitochondrial Dysfunction in Idiopathic and Monogenic Parkinson’s Disease

Parkinson’s disease (PD) is the second most common neurodegenerative disorder and affects millions worldwide [1]. Besides rapid progress in the elucidation of underlying disease mechanisms, no disease-modifying treatments are available today [2]. The underlying molecular mechanisms are complex and involve a plethora of interconnected pathways [3]. However, most likely due to their central role in cellular homeostasis, mitochondrial dysfunction has been identified to play a dominant role in PD onset and progression [4]. These insights were derived from environmental and genetic studies of mitochondrial dysfunction in PD, and overwhelming evidence has been gathered to support the hypothesis of mitochondrial dysfunction as a main driver of the disease. Several genes causing monogenic PD are either directly (*PRKN*, *PINK1*, and *DJ-1*) or indirectly (*GBA*, *LRRK2*, among others) linked to mitochondrial dyshomeostasis [5]. The deepened understanding of the monogenic forms of PD has already expanded our knowledge of disease mechanisms in idiopathic PD. Many shared pathways and pathophysiological overlap suggest mutual disease mechanisms [6]. Therefore, targeting mitochondrial dysfunction seems to be a tempting approach to develop innovative disease-modifying therapies [7].

In contrast, as a generic term, “mitochondrial dysfunction” oversimplifies the multi-faceted nature of mitochondrial biology in health and disease. PD-associated mitochondrial dysfunction presents with a variety of molecular events, including impaired mitochondrial biogenesis, increased release of reactive oxygen species (ROS), defective mitophagy and trafficking, electron transport chains (ETC) dysfunction, variations in mitochondrial dynamics, calcium (Ca^2+^) imbalance, neuroinflammation, and possible indirect influences on mitochondrial homeostasis from presumably unrelated pathways (e.g., α-synuclein deposition) [8]. The centrality of mitochondria in cellular functioning and the convergence of mammalian metabolism on mitochondria suggest that potential therapies must address a complex web of interconnected pathways [9]. Besides the complexity of mitochondrial dysfunction, all pathways, as mentioned above, share the common end route of impaired cellular bioenergetics (by altered oxidative phosphorylation, OXPHOS) This can result in an increase of ROS, which finally leads to cell death. Interestingly, primary mitochondrial disorders occasionally present with parkinsonism, e.g., in patients harboring mutations in the nuclear *POLG* gene [10]. Research has shown that PD patients have an increased somatic mitochondrial DNA (mtDNA) mutation load [11]. These findings have led to the discovery of mtDNA alterations as a pathophysiological driver of mitochondrial dysfunction in PD. Therefore, studies of mitochondrial disorders may help gain deepened insights into mitochondrial biology and pinpoint potential drug targets to enhance distinct aspects of mitochondrial biology [12]. Many dysregulated pathways in primary mitochondrial disorders are shared with PD: These pathways include OXPHOS deficiency, mtDNA maintenance defects, mitochondrial translation defects, and mitochondrial quality control defects, among others [13]. The discovery of genetic abnormalities in primary mitochondrial disorders may foster the identification of viable drug targets in PD. Even though this approach will need additional experimental validation, it may provide promising impulses for future studies.

### 1.2. The Scope of This Review

This narrative review highlights the importance of mitochondrial dysfunction as a molecular disease cause in monogenic and idiopathic PD. We will focus on gene therapeutic targets and challenges necessary to overcome to translate molecular findings into potential clinical applications. We will highlight different treatment strategies and evaluate their translational potential. In conclusion, we will define crucial knowledge gaps and molecular aspects where additional experimental validation is needed.

### 1.3. Current Gene Therapeutic Approaches in Parkinson’s Disease

The term “gene therapy” describes the delivery of a specific transgene to treat a given disease. The transgene either corrects or replaces a defective gene or supports cells in the diseased environment. Gene therapy vectors may be viral (commonly adeno-associated viruses (AAVs) and lentiviruses) or non-viral (typically naked DNA or in combination with cationic complexes or polymers) and can widely differ in their respective administration routes [14].

The term “gene therapy” is filled with immense hopes and will provide treatment options for so far untreatable diseases. However, the first gene therapies have only recently received formal approval for merely a few but constantly growing numbers of conditions. For PD, gene therapies are not yet available, but certain concepts currently undergo preclinical and clinical evaluation [15]. PD-related gene therapy in humans currently pursues three main directions:

1. Enhancement of dopamine synthesis by overexpression of relevant synthesis-related enzymes (tyrosinhydroxylase [*TH*], aromatic L-amino acid decarboxylase [*AADC*], GTP cyclohydrolase I [*GCH1*], or a combination thereof) [16].
2. The overexpression of neurotrophic factors (e.g., glial cell line-derived neu-rotrophic factor [*GDNF*] or neurturin [*NTN*]) [17].
3. The overexpression of glutamate decarboxylase [*GAD*] in the STN to decrease the synthesis of glutamate therein and to modulate basal ganglia loops in the human brain [18].

**Figure 1:**
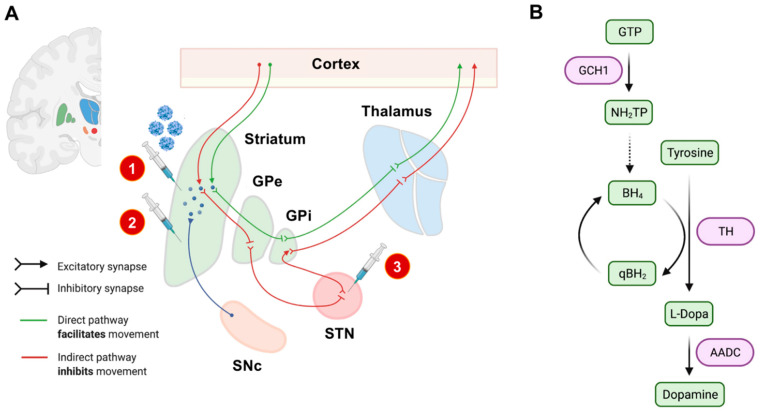
Experimental gene therapeutic approaches for the treatment of PD. In (**A**), neuroanatomical target regions and their functional interconnections are schematically depicted. So far, mainly two target sites have been evaluated: the striatum and the subthalamic nucleus (STN). Different treatment strategies and injection sites are highlighted by syringes. The red circled numbers refer to the numbered list of currently employed gene therapeutic strategies: 1. enhancement of dopamine production, 2. delivery of neurotrophic factors, and 3. overexpression of *GAD* to modulate basal ganglia loops. The SNc has not yet been evaluated as an injection site, mainly based on its limited accessibility by stereotactic surgery. In (**B**), we schematically highlighted the currently considered strategies for enhancing dopamine synthesis. The so-far investigated dopamine metabolism-related enzymes and their respective role in dopamine synthesis are highlighted in purple. AADC: L-amino acid decarboxylase. BH_4_: Tetrahydrobiopterin. GCH1: GTP cyclohydrolase I. GPe: external globus pallidus. GPi: internal globus pallidus. GTP: guanosine triphosphate. NH_2_TP: dihydroneopterin triphosphate. PD: Parkinson’s disease. eqBH_2_: quinoid dihydrobiopterin. SNc: substantia nigra pars compacta. STN: subthalamic nucleus. TH: tyrosine hydroxylase.

For these three approaches, the transgene has to be injected stereotactically in predefined neuroanatomical regions. The treatment is considered to be safe but appears to provide only limited clinical benefits for PD patients until now [19]. However, none of these approaches so far targets underlying pathophysiological traits of PD, and the applicability of neurotrophic factors to achieve relevant disease modification needs to be critically evaluated. Current experimental gene therapeutic strategies are highlighted in Figure 1.

### 1.4. A Primer on Mitochondrial Biology

Mitochondria are dynamic organelles and form a highly responsive network within every cell of the body [20]. Their primary role is to provide cellular energy via OXPHOS [21]. Mitochondria have two phospholipid membranes compartmentalizing distinct physiological functions of this organelle. The spatial and functional compartmentalization creates additional pharmacodynamic challenges for targeted drug delivery [22]. The outer mitochondrial membrane (OMM) is used to separate the organelle from the cytosol. The inner mitochondrial membrane (IMM) contains the necessary components for ATP synthesis via OXPHOS and separates the intermembrane space from the mitochondrial matrix. Here, the complexes I to IV of the ETC are used to create an electrochemical gradient (across the IMM). This gradient is used by the ATP synthase, also known as complex V, to generate ATP. As a result of OXPHOS, significant amounts of ROS are produced following this process [23].

In humans, more than 1500 genes encode the mitochondrial proteome [24]. The vast majority of these genes are encoded in the nuclear genome (nDNA), and their protein products are imported into the mitochondria following translation [25]. However, mitochondria also have their own genetic material (mtDNA, between 5 to 10 copies per mitochondrion) with only 13 proteins of the mitochondrial proteome being encoded in the mtDNA. In total, 37 genes are encoded in mtDNA, also including two mitochondria-specific ribosomal RNAs (rRNAs) and 22 transfer RNAs (tRNAs). These essential polypeptides are required for OXPHOS and are synthesized by mitochondrial ribosomes in the mitochondrial matrix [25]. Unlike the nDNA, multiple copies of the mitochondrial genome are present in one cell, mainly depending on the intracellular energy requirements. The number of copies of mtDNA depends on the cell type and tissue ranging between 1000 to 10,000 copies per cell [26].

Many of the imported (nDNA-encoded) proteins are critical for mtDNA-related functions such as transcription, maintenance, and translation [27]. At least 80 of these imported proteins shape the mitochondrial ribosomes specialized in mtDNA-encoded polypeptide synthesis. Because of their interdependence, coordinated regulation of nDNA and mtDNA gene expression is crucial to ensure cellular homeostasis and the satisfaction of tissue-specific energy needs [28]. There are essential differences between the nDNA and the mtDNA genetic codes, e.g., the triplet UGA codes for tryptophan in mtDNA and act as a stop codon in nDNA. In general, mtDNA is highly susceptible to mutations, and there are often two (or more) populations of mtDNA present in one cell, a phenomenon called heteroplasmy [25]. These mtDNA mutations and their degree of heteroplasmy can either be inherited by maternal transmission or can occur spontaneously due to somatic mutations [29]. In contrast to nDNA, mtDNA replication is highly error-prone, and mitochondria only have limited DNA repair mechanisms [30].

As mtDNA is randomly separated during cell division and mitotic separation, the percentage of different mtDNA populations in cells and tissues might substantially differ in daughter cells [31]. In addition, somatic mutations naturally occur over time, and mutated mtDNA genomes can build up, particularly in postmitotic tissues like the brain [32]. The respective proportion of mutated DNA in a given tissue determines the phenotypic expression of mitochondrial dysfunction. For example, to alter OXPHOS, a minimum amount of mutated mtDNA must be present in a particular tissue. However, the relevant thresholds for a given cell population to suffer from mitochondrial dysfunction are widely unknown [33]. Most likely, high energy-demanding tissues (such as the neurons of the CNS) require lower thresholds of mutated mtDNA to result in bioenergetic depletion. Depletion of mtDNA can also cause disrupted mtDNA protein synthesis and thus lead to insufficient energy production in the affected cells [34]. Furthermore, nDNA variation could be mainly responsible for these mtDNA changes, given the importance of nDNA-encoded genes in mtDNA-related processes [35]. Depending on the degree of heteroplasmy and the localization of specific mtDNA mutations, any change can lead to mitochondrial dysfunction and subsequent cell death [36].

### 1.5. Parkinson’s Disease as a “Mitochondrial DNA Maintenance Disorder”

#### 1.5.1. Mitochondrial DNA Changes in Aging and Neurodegeneration

mtDNA substantially differs from nDNA. mtDNA is organized in circles and does not undergo any condensation (i.e., caused by the absence of histones). It is, therefore, less protected against any mutagenic agents, such as ROS, which naturally occurs close to the mitochondrial genome [13]. It is also more vulnerable to any enzymatic disruption or spontaneous hydrolytic processes [37]. Besides, mitochondria are not capable of the same level of quality for DNA repair and undergo more error-prone DNA replication steps [30]. These circumstances lead to approximately ten times higher mutation rates in the direct comparison of mtDNA to nDNA [27]. In general, pathological modifications of the mitochondrial genome can be divided into three main groups:

1. mtDNA point mutations (either inherited or somatic mutations),
2. mtDNA deletions, and
3. an overall reduction of mtDNA copy numbers [38].

**Figure 2:**
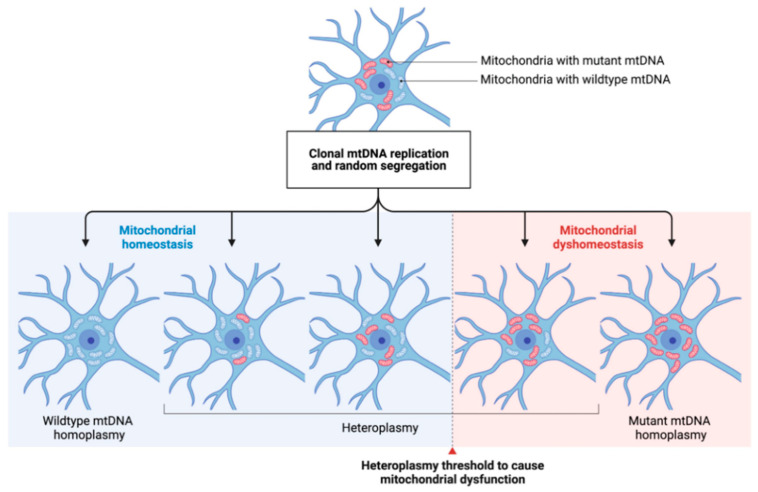
The concept of mtDNA heteroplasmy and its contribution to mitochondrial dysfunction in PD. The arbitrary occurrence of mtDNA mutations (depicted by red-colored mitochondria) and their respective clonal expansion leads to different degrees of heteroplasmy in a given neuronal cell. Cell-specific heteroplasmy thresholds (dashed line) to cause mitochondrial dysfunction are widely unknown. By, e.g., shifting the degree of heteroplasmy towards a higher ratio of wildtype/mutated mtDNA, mitochondrial homeostasis might be restored. mtDNA: mitochondrial DNA.

Both, point mutations and mtDNA deletions are subject to clonal expansion. As mitochondria replicate independently from the cell cycle and distribute randomly to the daughter cells after mitosis, the degree of heteroplasmy can widely differ within a given tissue [39]. If a certain heteroplasmy threshold is exceeded, mitochondrial homeostasis can be impaired, subsequently leading to impairments similar to those seen in primary mitochondrial disease and ultimately to cell death [40] (see Figure 2). There is strong experimental evidence that genetic variations in mtDNA increase with age, which also translates to our pathophysiological understanding of the development of neurodegenerative diseases [41]. It has also been shown that the mtDNA mutation rate accelerates with higher age which is especially relevant to postmitotic neurons [42].

#### 1.5.2. Inherited and Somatic mtDNA Point Mutations and Their Role in the Pathophysiology of Parkinson’s Disease

Inherited mtDNA point mutations are of negligible relevance for the vast majority of PD cases. However, Shoffner et al. (1993) described a point mutation (m.1555A>C., *MT-RNR1*) within the 12S-rRNA gene in a pedigree of maternally transmitted hearing loss and levodopa-responsive parkinsonism [43]. In another pedigree, the heteroplasmic mtDNA point mutation m.1095A>C in the *MT-RNR1* gene has been identified as a potential cause of PD [44]. The latter is especially intriguing as it impairs the complex I function of the ETC, a pathophysiological hallmark often observed in PD [44]. These findings were supported by the later identification of additional mtDNA missense mutations present in nearly all mtDNA-encoded subunits of complex I [45]. Collectively, inherited mtDNA variants, referred to as mtDNA haplogroups, are associated with a lower or higher risk of developing PD [46,47,48]. Many of these reports need additional experimental validation. In summary, there is no direct evidence to suggest that inherited mtDNA point mutations are a primary cause of PD [49]. One study examined the combined mutational burden of somatic mtDNA point mutations in all genes encoding complex I subunits in postmortem PD brain tissue [37]. The authors concluded that there was no significant difference in the overall number of mtDNA point mutations in PD patients and controls.

In contrast, this work revealed relatively low levels of somatic mtDNA point mutations within the *MT-ND5* gene exclusively observed in idiopathic PD patients. However, the heteroplasmy thresholds were generally less than 1%, where no functional consequences would be expected [37]. The conflicting experimental data so far also points toward potential challenges for developing mitochondria-targeted gene therapies: Somatic mutations (deletions and/or point mutations) occur randomly by means of heteroplasmy and localization within the mitochondrial genome [32]. As the pathophysiological role of inherited mtDNA mutations is still under debate, the arbitrary occurrence of somatic mtDNA mutations complicates the design of mitochondrial genome editing techniques for this purpose. In addition, it is unclear how to define functionally relevant cumulative thresholds based on the simultaneous presence of different kinds and respective frequencies of somatic mtDNA mutations.

#### 1.5.3. The Role of Mitochondrial DNA Deletions and Copy Number Variations in PD

The most frequent deletion in human mtDNA encompasses ca. 5 kbp. This deletion includes most of the complexes of the ETC, leading to an overall bioenergetic deficit [43]. While it is not entirely understood how mtDNA deletions occur, several hypotheses were suggested: In most cases, mtDNA deletions occur randomly and appear to undergo clonal expansion. Another theory suggests that critical pathways for mtDNA replication and quality control are impaired in neurodegenerative disorders [40]. The maintenance of mtDNA requires a variety of nDNA-encoded gene products. The proteins involved in mtDNA replication have been termed replisome [50]. The mtDNA replisome consists of the mtDNA polymerase γ (a complex of *POLG* and *POLG2* gene products), the mitochondrial transcription factor (*TFAM*), the DNA helicase twinkle (*TWNK*), and the mitochondrial single-stranded binding protein (*mtSSB*) [50]. Remarkably, variants in *POLG*, *TWNK*, and *TFAM* are not only known as a monogenic cause of primary mitochondrial disorders (occasionally presenting with parkinsonism) but can also increase the risk for PD [51]. Based on the known function of the mitochondrial replisome, mutations in these three genes can result in mtDNA deletions and decreased mtDNA copy numbers [49]. All three genes show high expression levels in neuroanatomical key structures involved in PD disease development such as the substantia nigra (SN) [51]. Postmortem studies also revealed lower levels of mtDNA transcription factor *TFAM* in the SN of PD patients [52]. In this study, *TFAM* and *TFB2M* levels correlated with decreased expression levels of complex I. Noteworthy, decreased mtDNA copy numbers showed a cell-specific distribution in PD [53]. In contrast to dopaminergic neurons of the SN, cholinergic neurons isolated from PD brains were associated with a higher mtDNA copy number [54]. It is also worth mentioning that many of the known monogenic PD genes (e.g., *PRKN* or *LRRK2*) have been linked to altered mtDNA maintenance [55,56,57]. However, future studies are needed to fully understand the interconnectedness of mtDNA maintenance and their impact on monogenic and idiopathic PD.

Even though mtDNA alterations have been observed in physiological aging, the increased amount of mtDNA rearrangements and deletions in PD patients suggest a certain disease specificity [58]. Accordingly, SN-related mtDNA deletions and copy number variations are more common in PD than in patients with other neurodegenerative diseases (e.g., Alzheimer’s disease, AD) [41]. PD patients are thus more likely to accumulate mtDNA mutations, particularly in dopaminergic neurons. Therefore, regulation of mtDNA deletions and copy number variations seems to be a potential mechanism to protect SN neurons from cell death or apoptosis.

Additional experimental evidence originates from animal models. A conditional *TFAM* knock-out mouse (MitoPark mouse) is characterized by respiratory chain deficiencies and low neuronal cell counts including progressive loss of dopaminergic neurons in the SN [59,60,61]. In another mouse model, mutant *TWNK* has been expressed in CNS neurons, leading to an increase of age-related mtDNA deletions and dopaminergic neurodegeneration [62]. These mice suffer from levodopa-responsive motor impairment and show phenotypic features of premature aging. This data stresses that the integrity of the nuclear and the mitochondrial genome is critical for the survival of dopaminergic neurons.

The deepened understanding of mtDNA defects in PD may offer the opportunity for targeted therapies: mtDNA deletions in individual SN neurons can activate compensatory mechanisms mainly by triggering mitochondrial biogenesis [63,64]. These mechanisms increase the number of mtDNA copies, the formation of cristae networks, and dopamine synthesis. The compensatory response could be impaired or dysregulated by nDNA variants in the genes mentioned above and may impact PD onset and progression [35]. By employing compensatory mechanisms, individual neurons can overcome the harmful effects of mtDNA mutations below a certain threshold [64]. The increase of mtDNA copy numbers with a corresponding rise in wild-type mtDNA might therefore prevent respiratory chain defects in people with a high mtDNA deletion burden. Therefore, the enhancement of mitochondrial biogenesis could be specifically targeted by gene therapy to combat the unspecific accumulation of mtDNA mutations in PD patients [65].

## 2. Main Body

### 2.1. Defining Neuroanatomical Treatment Targets

The treatment of mitochondrial dysfunction in PD comes with unique challenges and opportunities. To date, it is unclear which brain regions are especially vulnerable to mitochondrial dysfunction. Most post-mortem studies focus on the SN as a target region of PD pathophysiology [66]. The neurodegeneration of the dopaminergic neurons in the SN has been postulated as a histopathological hallmark of PD [67]. Besides, the co-occurrence of impaired dopamine metabolism, the emergence of oxidative stress, and mitochondrial dysfunction have been proposed as a vicious cycle, self-amplifying the molecular roots of neurodegenerative processes in this disorder [68,69]. However, mitochondrial dysfunction is not restricted to dopaminergic neurons but also affects other neuronal and non-neuronal cell populations. This idea is additionally supported by our current understanding of PD as a network disease, affecting widespread areas of the human brain [70]. Whether spatially non-specific drug delivery to the CNS or targeted intraparenchymal drug delivery in predefined neuroanatomical regions (e.g., the basal ganglia) will be the most promising approach in the future, is still under debate [15]. However, the concept of spatial drug delivery does not only concern distinct neuroanatomically defined regions [71]. There is a close metabolic interconnectedness between glial cells and neurons, and mitochondrial dysfunction most likely extends to several CNS cellular subpopulations [72]. The spatial complexity is not the only challenge concerning drug delivery. Different treatment strategies are discussed in the following. Based on the complex intracellular compartmentalization of mammalian cells, it is vital to consider whether gene therapeutic approaches target the nucleus or the mitochondrial matrix. These different levels of spatial complexity substantially aggravate anyhow preexisting challenges for CNS-based drug delivery (e.g., overcoming the blood-brain barrier (BBB)) [73].

In general, two common approaches for drug delivery are available: direct and indirect CNS delivery [74]. Direct CNS drug delivery describes the administration route via intraparenchymal application (e.g., by stereotactically placed catheters, similar to the procedure for electrode implantation for deep brain stimulation) [74]. This term also extends to intrathecal, intracerebroventricular, and subpial administration. In contrast, indirect CNS drug delivery describes the administration via an intravenous infusion [75]. An additional supportive method is the transient opening of the BBB by focused ultrasound which has, however, not yet been clinically evaluated [76]. The concept of cellular tropism (by means of tissue-/cell type-specificity) can be achieved by employing different Adeno-associated virus (AAV) serotypes and respective transgene designs (e.g., by using cell-specific promoters) [77]. If the development of targeted liposomes can achieve identical (pre-)clinical efficacy to AAV-based methods, will be the subject of future studies [78].

AAVs are great candidates for gene therapy because of their low risk of insertional mutation and long-term persistence within cells [79]. Currently, AAV-based approaches are the central concept for gene therapy in preclinical and human use. AAVs are non-pathogenic in humans but may induce immunological host responses, potentially hindering their long-term use in a given patient. AAV-based vectors can be fine-tuned by specific capsid and promotor designs. Research has shown that distinct virus strains show a relatively specific tissue tropism [79].

The payload of gene therapies can be specifically designed to meet molecular needs: This can be achieved by the overexpression of genes by gene replacement (e.g., for loss-of-function mutations), the silencing of genes by small hairpin RNA (shRNA), or small interfering RNA (siRNA) (e.g., for gain-of-function mutations), site-directed genome editing (in general suitable for a variety of mutation types), and the modulation of gene expression by microRNAs (miRNAs) or modified genome editing technologies [17,80]. However, most of these approaches have not made their way into clinical use. In addition, for safe and effective gene therapy, specific gene regulatory elements can be chosen to achieve cell-specificity (e.g., by cell-specific promoter sequences) [79]. When choosing a target or disorder to pursue gene therapy, it is crucial to consider the possibility of successfully delivering an AAV vector into the CNS.

### 2.2. Treatment Strategies

The development of gene therapies for neurological disorders is a highly dynamic field of research, and the last years have shown impressive advances. However, gene therapeutic approaches targeting mitochondrial dysfunction in neurodegenerative diseases are currently sparse, even in pre-clinical phases. This may be due to the significant challenges mitochondrial gene therapy encounters in vivo, many caused by the complex mitochondrial biology [81]. In general, the combination of different gene therapies would likely be the most efficient treatment strategy, mainly due to the interwovenness of the nuclear and mitochondrial genome, unique characteristics of mtDNA, such as high mtDNA copy numbers, heteroplasmy, and the mtDNA-specific genetic code. These limitations must be addressed before mitochondrial gene therapy can be used effectively in the context of PD. In this review, we will describe specific challenges for mitochondrial gene therapy and will focus on four potential therapeutic strategies:

1. gene replacement/correction of monogenic PD genes,
2. gene replacement of nuclear-encoded mitochondrial genes,
3. allotopic expression of mtDNA-encoded genes, and
4. mtDNA genome editing.

#### 2.2.1. Gene Therapies of Monogenic Parkinson’s Disease Genes to Treat Mitochondrial Dysfunction

The discovery of monogenic PD genes has led to in-depth insights into relevant disease mechanisms, broadening our molecular understanding of idiopathic PD [82]. Previously, mitochondrial dysfunction has already been implicated in idiopathic PD cases based on environmental studies [83]. The discovery of the *PRKN* and *PINK1* genes has grounded the concept of mitochondrial dysfunction on a genetic basis [4].

Mutations in both genes are inherited in an autosomal recessive fashion. Truncated or missense variants of the *PRKN* or *PINK1* gene have been shown to result in a loss-of-function or complete inactivation of their respective gene products. Later studies have demonstrated that *PRKN* and *PINK1* work together in a shared pathway and are mainly responsible for mitochondrial quality control by removing dysfunctional mitochondria (a process called mitophagy) [84]. Dysfunctional Parkin or PINK1 leads to impaired clearance of damaged mitochondria [85,86]. Intracellularly, damaged mitochondria can present with any aspects of mitochondrial dysfunction, including OXPHOS deficiency and impaired mtDNA maintenance [43]. Another monogenic PD gene that has been directly linked to mitochondrial dysfunction is *DJ-1* [87,88].

Even though the precise function of DJ-1 remains unclear, it is thought of as an oxidative stress sensor and works synergistically together with *PRKN* and *PINK1*. *PRKN*, *PINK1*, and *DJ-1* are the most prominent examples of monogenic PD genes directly leading to mitochondrial dysfunction [89,90]. However, most of the other identified monogenic PD genes (e.g., *SNCA* or *LRRK2*) have also been experimentally associated with mitochondrial dysfunction [91,92,93,94]. Whether mitochondrial dysfunction is a cause or consequence of neurodegenerative processes in other monogenic PD forms will need additional experimental validation.

Nonetheless, overexpression or silencing (depending on the relevant mutation type) of other monogenic PD genes can present viable treatment targets for improving mitochondrial dysfunction in monogenic PD [4]. Many of these treatment strategies may also extend to idiopathic PD. We kindly refer the reader to the review by Bloem et al. [1].

Previous studies have shown that *PRKN* overexpression can protect against cellular insults directed against mitochondria [95,96,97,98]. For example, the overexpression of wild-type *PRKN* in transgenic mice models reduced 1-Methyl-4-phenyl-1,2,3,6-tetrahydropyridine (MPTP)-induced (a known inhibitor of complex I) mitochondrial damage and prevented dopaminergic neurodegeneration [99]. The intranigral AAV-based delivery of wild-type *PRKN* prevented motor impairments and dopaminergic cell loss in a chronic MPTP minipump mouse model [100]. In Drosophila flies, the knockout of the *PINK1* homolog can lead to male sterility and progressive muscle wasting [101]. Here, defects in mitochondrial structure and increased sensitivity to oxidative stress can be observed. The Drosophila *PINK1*-KO phenotype can be rescued by overexpression of human *PINK1* [95]. The overexpression of *PRKN* has been shown to rescue mutated *PINK1* phenotypes, most likely by Miro-mediated phosphorylation and subsequent proteasomal degradation of dysfunctional mitochondria [102,103]. Furthermore, overexpression of *PINK1* has also been shown to rescue the α-synuclein-induced phenotype in Drosophila [104,105]. Additional evidence can be derived from siRNA experiments. Here, *PINK1*-silencing caused neuronal toxicity, which has been aggravated by MPTP administration in mice [106]. The wild-type but not the mutated form of *PINK1* protected neurons against MPTP-mediated cell death. The AAV-mediated expression of *PRKN* or *DJ-1* can protect mitochondria of dopaminergic neurons, even when *PINK1* is absent [106]. An overview of monogenic PD genes and their respective link to mitochondrial dysfunction is highlighted in Table 1.

#### 2.2.2. Gene Repair and Enhancement of Nuclear-Encoded Mitochondrial Genes

AAV-mediated gene therapies have been tested in different models of primary mitochondrial diseases [107]. Insights derived from primary mitochondrial disorders elucidated potential gene therapy targets to treat mitochondrial dysfunction in PD. These strategies extend to the repair or enhancement of non-classical monogenic PD genes. In this context, we have already discussed “mtDNA maintenance disorders”, which can clinically present with parkinsonian features, and stressed the role of increased somatic mtDNA mutations in the onset and progression of PD [10]. Gene therapy-based replacement (if there is a disease-causing mutation in patients present) or enhancements (by overexpression of genes even in the absence of a disease-causing mutation therein) of the mtDNA replisome may be helpful to improve mtDNA maintenance. Based on previous studies, the most promising genes for this approach are *POLG*, *POLG2*, *TWNK*, and *TFAM* [51]. Experimental evidence can be derived from the transfection of *TFAM*. Here, PD-derived nigral cybrid cell lines (cell lines that incorporate the nuclear genome from one cell with the mitochondrial genome from another cell) can restore mitochondrial bioenergetics by overexpression of *TFAM* [108]. However, it is unlikely that all PD patients show a marked increase in mtDNA damage at a given time. If nDNA mutations in the given genes are present in a patient, different gene therapies treatment strategies (e.g., gene editing by CRISPR/Cas9, Clustered Regularly Interspaced Short Palindromic Repeats/CRISPR-associated protein 9) could also be experimentally evaluated [80].

**Figure 3:**
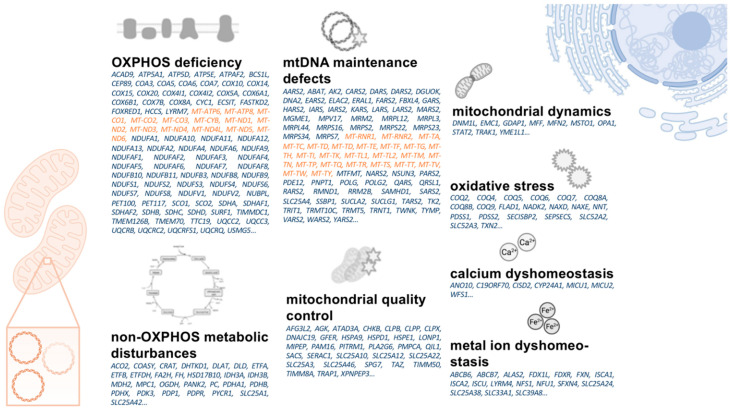
Overview on mtDNA- (orange) and nDNA- (blue) encoded genes causative for primary mitochondrial disorders. The listed genes are ordered based on pathophysiological concepts their respective disorders share with aspects of mitochondrial dysfunction in PD. mtDNA: mitochondrial DNA. nDNA: nuclear DNA. OXPHOS: oxidative phosphorylation. PD: Parkinson’s disease.

Many potential target genes can be identified from primary mitochondrial disorders caused by nDNA-encoded genes [107]. The large number of nDNA defects leading to primary mitochondrial diseases helps to understand the many-faceted nature of mitochondrial dysfunction [5]. Even though about 300 nDNA genes have been suggested to be associated with primary mitochondrial disorders, their gene products only account for a distinct subset of the overall mitochondrial proteome [107]. However, it is necessary to prioritize treatment targets, and the nDNA mutations causing primary mitochondrial diseases represent a reasonable starting point for treating other disorders. In addition, functional data are available for many causative nDNA genes and have been linked to distinct partial aspects of mitochondrial dysfunction. For example, *DNM1L*, *GDAP1*, *MFF*, *MFN2*, *MSTO1*, *OPA1*, *STAT2, TRAK1*, and *YME1L1* cause primary mitochondrial diseases mainly by impacting mitochondrial dynamics [107]. Disruption of mitochondrial dynamics has already been proposed in the pathophysiology of PD [109]; therefore, it is reasonable to assume that influencing the aforementioned genes may help treat this aspect by altering their respective gene expression. Prioritizing treatment targets helps substantially to streamline the drug development pipeline. In combination with high-throughput methods, potentially positive treatment effects can be validated in a reasonable time frame [34]. Figure 3 provides an overview of mtDNA- and nDNA-encoded genes causative for primary mitochondrial diseases as potential therapeutic targets in PD.

#### 2.2.3. Allotopic Expression of mtDNA-Encoded Mitochondrial Genes

Mutations within the mtDNA appear to accumulate randomly in PD patients over time. The lack of mtDNA quality control and repair systems leads to the assumption that “allotopic expression” of mtDNA can be an approach in treating mitochondrial dysfunction [110]. The term “allotopic” means that mtDNA-encoded genes are either transiently expressed in the nucleus or permanently inserted in the non-coding regions of the nuclear genome [111]. In general, the allotopic expression strategy was developed to treat primary mitochondrial diseases caused by mtDNA mutations. The design of allotopically expressed mtDNA genes must adhere to different design standards: a mitochondrial targeting sequence is necessary so that the encoded protein is directed to the mitochondria. Additionally, differences in the codons used by the nuclear and mitochondrial genomes and differing codon preferences between the nuclear-cytosolic and mitochondrial translation systems must be considered [112]. mtDNA-encoded genes include 13 ETC complex subunits (for complex I, III, IV, and V), 22 mitochondria-specific tRNAs, and two mitochondrial rRNAs [24]. Based on the random emergence of mtDNA variants in PD, all mtDNA-encoded genes could potentially represent valuable treatment targets. However, it is likely that OXPHOS deficiencies, as the hallmark and common end route of mitochondrial dysfunction, should be prioritized for drug development [113].

The concept of allotopic expression gene therapies is currently being tested in humans. A phase I/II clinical trial aims to treat Leber’s hereditary optical neuropathy (LHON) by intravitreal injection and allotopic overexpression of the mtDNA-encoded NADH:ubiquinone oxidoreductase (complex I) (NCT04912843). The NADH:ubiquinone oxidoreductase can also be a viable treatment target in PD, where complex I deficiency has been repeatedly reported [44]. Whether a combined allotopic expression of mtDNA-encoded ETC complexes can provide additional therapeutic benefits needs additional experimental validation. Low vector capacities may additionally hinder the combined allotopic expression of mtDNA genes [79]. There are still a few challenges to accelerate the application of allotopic gene expression. These include necessary improvements in nuclear gene expression, mitochondrial import of cytosolic proteins, posttranslational protein modifications, and functional integration in mitochondrial protein complexes [114,115,116].

#### 2.2.4. Mitochondrial DNA Genome Editing and Heteroplasmy Shifting

Most pathogenic mtDNA mutations require a critical threshold to cause harm to cells. This aspect has been employed as a potential treatment paradigm named heteroplasmy shifting [117,118]. The underlying idea is to decrease the cumulative amount of mutated mtDNA below a disease-causing heteroplasmy threshold. To achieve this goal, various genome editing methods have been modified to alter mtDNA sequences in a targeted and predictable manner [119]. These methods included mitochondria-targeted restriction endonucleases, zinc finger endonucleases (ZFNs), transcription activator-like effectors nucleases (TALENs), and the CRISPR/Cas9 methodology [118]. Many of these methods have already been employed in the preclinical treatment of primary mitochondrial diseases. For example, the restriction endonuclease SmaI decreased the mutation load in cybrid cell lines with the m.8399T>G mutation causing neuropathy, ataxia, and retinitis pigmentosa (NARP syndrome) [120]. Subsequent functional analyses revealed an increase in ATP levels following genome editing in these cell lines. These findings have also been confirmed in heteroplasmic mouse models of primary mitochondrial diseases following AAV transfection of restriction endonucleases [121].

However, suitable restriction sites are limited in mtDNA, so more flexible approaches have been designed. These limitations can potentially be overcome by introducing programmable nucleases like ZFNs [122] or TALENs [123,124]. These methods, widely known from nuclear genome editing, have been specifically modified to be employed in mtDNA genome editing (mtZFNs and mitoTALENs). Even though these methods have been successfully applied in the preclinical evaluation for primary mitochondrial disorders, the clinical use can be limited by inducing rapid mtDNA depletion in humans [125]. This can mainly be caused by the lack of suitable mtDNA repair mechanisms following the double-strand breaks introduced by these two methods [27]. Interestingly, the rise of CRISPR/Cas9 technology for nuclear genome editing faces significant challenges in mitochondrial genome editing: the import of sgRNA (single guide RNA, the relevant functional component of CRISPR/Cas9 for site-directed specificity) is generally limited by poor RNA import capabilities of mitochondria [119]. Even though mitochondrial genome editing and subsequent heteroplasmy shifting will likely be a viable approach for treating distinct primary mitochondrial disorders (caused by single-site mtDNA point mutations), the applicability in PD is unlikely. Because of the random appearance of multiple mtDNA point mutations, a specific site targeted design will be nearly impossible. However, extensive research is needed to evaluate whether single mtDNA point mutations in PD occur with a higher frequency to define potential treatment targets. Our knowledge in this area is still limited at the moment.

### 2.3. Special Considerations for Mitochondrial Gene Therapy

Allotopic expression of mtDNA faces significant challenges: each allotopic expressed mtDNA gene needs to be imported to the mitochondria via a complex mitochondria import machinery consisting of various proteins [126]. This is achieved most easily by attaching a mitochondrial targeting sequence to the allotopic expressed mtDNA gene [127]. It effectively leverages the mitochondrial import machinery to bring the respective protein to the correct mitochondrial compartment. Unfortunately, the import of allotopic expressed mtDNA-encoded proteins may not be as simple of a solution as it initially appears. Recent research has shown that the overproduction of nDNA-encoded and allotopically expressed mtDNA proteins can itself cause mitochondrial dysfunction [128]. Producing defective or misfolded mitochondrial protein precursors from the nuclear genome can cause a toxic build-up in the cytosol. This unphysiological high expression level of mitochondrial proteins can be named “mitochondrial protein import stress”. The build-up accumulation of misfolded protein in the mitochondria can cause severe disruption of OXPHOS, proteotoxic stress, and mtDNA depletion [129]. Mitochondrial protein import stress can provide a tremendous challenge for gene therapies targeted against mitochondrial dysfunction [130]. For example, the subunits of ETC complexes to ensure efficient OXPHOS is highly regulated and kept in a balanced equilibrium state. By tipping over this fine-tuned balance, disassembled ETC complexes can lead to impaired OXPHOS and bioenergetic depletion [131]. The subsequent increase of ROS by impaired OXPHOS can further damage mitochondrial and overall cellular structures paving the way into a vicious cycle. Based on the additional presence of heteroplasmy, this situation can become highly complex and unpredictable concerning the design of mitochondrial gene therapies.

In summary, we have discussed several challenges for the experimental and clinical evaluation of mitochondrial gene therapies. It is necessary to identify PD patients with clear-cut mitochondrial dysfunction. There are currently no established methods for patient stratification. Most likely, not all patients with PD primarily suffer from disease-relevant mitochondrial dysfunction at any given time. Identifying a window of opportunity for treatment will be one of the significant challenges for successful clinical trial designs. Extensive longitudinal data is needed, in particular in the prodromal stage of PD. However, reliable biomarkers to achieve this goal have not yet been concludingly established [132]. A promising approach can derive from enhanced insights into the individual disease genetics (e.g., by presymptomatic genetic testing or polygenetic risk scoring) [133]. However, blood- or neuroimaging-based assessments of mitochondrial dysfunction can substantially enhance our current understanding of mitochondrial dysfunction’s temporal and spatial dynamics in vivo [8]. Current knowledge gaps of essential aspects of mitochondrial biology need to be closed for the rational design of gene therapies. This aspect extends to unclear elements of the mitochondrial import machinery, our incomplete understanding of mitochondrial protein import stress, and unknown tissue-specific mtDNA heteroplasmy thresholds. Treatments targeting the mitochondrial genome require specific genome editing techniques. Drug delivery to the mitochondrial matrix can be substantially hindered by the subcellular compartmentalization and respective physical barriers to be overcome (BBB, cell membranes, OMM, and IMM).

## 3. Conclusions

Mitochondria-targeted gene therapies may offer potential possibilities in the treatment of PD. The recent progress in gene therapy-based treatment strategies for primary mitochondrial disorders is relevant to understanding the potential use in PD and other neurodegenerative diseases [134]. However, particular challenges need to be overcome and additional research is required to broaden our understanding of mitochondrial biology in PD. Delivery methods must consider the specific properties of the specific mitochondrial proteins, including their location within the mitochondrial organelle, and how they will be targeted there without overwhelming the mitochondrial import machinery. General advancements of gene therapy (e.g., genome editing technologies) will benefit the development of innovative treatment strategies. Prioritization of drug targets and sophisticated design strategies are needed to ensure the subsequent success of gene therapy in clinical trials. These challenges are not insurmountable, but remarkable knowledge gaps need to be closed before PD patients may benefit from such potentially disease-modifying treatments.

## Figures and Tables

**Table 1:** Established causative genes of monogenic PD and their respective association with mitochondrial dysfunction.

Gene Name	Mode of Inheritance	Parkinson’s Disease Phenotype	Mitochondrial Involvement in Disease Pathophysiology–Key Mechanisms	References
*ATP13A2* *(PARK9)*	AR	Atypical PD, Kufor-Rakeb syndrome	Impaired mitochondrial clearance, mitochondrial dysfunction due to zinc dyshomeostasis	Ramirez et al., 2006 Grunewald et al., 2012 Park et al., 2014
*DJ-1* *(PARK7)*	AR	Early-onset PD	Reduced anti-oxidative stress mechanisms	Bonifati et al., 2003 Takahashi-Niki et al., 2004
*FBXO7* *(PARK15)*	AR	Atypical PD	Aggravated protein aggregation in mitochondria, impaired mitophagy	Shojaee et al., 2008 Zhou et al., 2018
*GBA*	AD	resembling IPD with more rapid cognitive and motor progression, dementia with Lewy bodies	Impaired mitophagy	Sidransky et al., 2009 Barkhuizen et al., 2016 Zhao et al., 2016 Gegg et al., 2016 Moren et al., 2019
*LRRK2* *(PARK8)*	AD	resembling IPD	Disturbance in mitochondrial ATP and ROS production, impaired mitochondrial dynamics and mitophagy, mitochondrial DNA damage	Zimprich et al., 2004 Mancini et al., 2020
*PINK1* *(PARK6)*	AR	Early-onset PD	Defective mitochondrial quality control	Valente et al., 2004 Ge et al., 2020
*PLA2G6* *(PARK14)*	AR	Atypical PD, NBIA type 2B, Infantile neuroaxonal dystrophy 1	Maintenance of mitochondrial function, impaired mitophagy	Paisan-Ruiz et al., 2009 Chiu et al., 2017 Chiu et al., 2019
*PRKN* *(PARK2)*	AR	Early-onset PD	Defective mitochondrial quality control	Kitada et al., 1998 Ge et al., 2020
*SNCA* *(PARK1)*	AD	May be atypical (higher frequency of cognitive/ psychiatric symptoms)	Mitochondrial toxicity, fragmented mitochondria	Polymeropoulos et al., 1997 Singleton et al., 2003 Chartier-Harlin et al., 2004
*VPS35* *(PARK17)*	AD	resembling IPD	Regulation of mitochondrial dynamics and homeostasis	Vilarino-Guell et al., 2011 Zimprich et al., 2011 Cutillo et al., 2020

AD: autosomal dominant. AR: autosomal recessive. IPD: idiopathic Parkinson’s disease. NBIA: neurodegeneration with brain iron accumulation. PD: Parkinson’s disease. The table has been adapted and modified from Prasuhn et al. [8].

## Data Availability

Not applicable.
